# “Loops and hurdles”: secondary analysis of patient interview data to explore the experience of patient access to UK general practice

**DOI:** 10.1186/s12875-026-03376-5

**Published:** 2026-05-16

**Authors:** Lucy Frost, Wahab Alazawi, Chloe Phillips, Abi Eccles, Catherine Pope, Helen Atherton

**Affiliations:** 1 Nuffield Department of Primary Care Health Sciences, University of Oxford, Oxford, UK; 2 Primary Care Research Centre, University of Southampton, Southampton, UK

**Keywords:** Primary Care, General Practice, Access to Primary Care, Patient satisfaction

## Abstract

**Background:**

Many countries are experiencing a shortage of general practitioners, increased demand for primary care, resulting in difficulties for patients seeking access and leading to increased dissatisfaction. The NHS Delivery Plan for Recovering Access to Primary Care in May 2023 signalled a UK policy commitment to improve GP access.

**Methods:**

To explore patients’ experience of access to UK general practice as reported in previously conducted interview studies of COVID and non-COVID related conditions a secondary analysis of interviews with 286 participants from five qualitative interview studies originally carried out between 2020 and 2023 was conducted. Qualitative thematic secondary analysis using a mix of deductive and inductive coding was used to examine interview data about patients’ experiences of health conditions and health services.

**Results:**

The interview data reveal ‘loops’ and ‘hurdles’ experienced by patients which delay and complicate the process of accessing general practice. Obstacles to access included needing to phone back several times, using online triage systems and negotiating with receptionists. Some patients were able to develop strategies or had resources including time and knowledge of the health system that helped them overcome access barriers, but the overall picture is of difficult and often negative access experiences.

**Conclusion:**

Secondary analysis of archived health experience interview data can provide additional evidence about access to care. Policy and practice solutions to access challenges increasingly rely on the use of phone and online systems of triage and for appointment making but these can increase delays and are experienced as significant barriers to access, fuelling dissatisfaction with primary care.

**Supplementary Information:**

The online version contains supplementary material available at 10.1186/s12875-026-03376-5.

## Introduction

Primary care services are a core component of effective healthcare systems and the general practitioner (GP) or family doctor/physician plays a vital role as the first point of patient contact, resolving the majority of cases and referring on to specialist care if needed. However, many countries are experiencing a shortage of GPs/family physicians, alongside rising demand, resulting in difficulties for patients seeking primary care [1, 2]. The most recent UK government 10 year plan for the health service sought to directly address the ‘8am rush’ when patients try to access an appointment, by setting out policy commitments to improve access through expanded use of digital triage systems, multidisciplinary teams and alternative routes to care [3]. Difficulties getting an appointment with a GP or family doctor are significant in the UK and other countries, and the organisation and funding of primary care systems varies internationally and this shapes access challenges in different ways. Access is a significant source of dissatisfaction with health services in the UK [4] and elsewhere, for example in Canada where “less than half of Canadians are able to see a primary care provider on the same or next day” [5].

Understanding patient experience is key to understanding access challenges in general practice. This is especially crucial when policy responses increasingly emphasise efficiency, diversion and digital solutions, which may not align with patients’ experiences of access or equity objectives [6]. Research and practice has traditionally focused on interventions to reduce demand, at the cost of considering patient related outcomes [7] but qualitative approaches offer a way to understand in more depth how patients experience healthcare and access to it.

As part of a wider project focused on access to general practice [6], we sought to augment our understanding of patient experience by exploring perspectives of those engaged with the health service, by conducting a secondary analysis of interview data. While secondary analysis of qualitative interview data has traditionally been less common than secondary analysis of quantitative data, there is growing interest in re-using data from previous research to address new research questions and inform policy [8]. This paper addresses the question “what can patient accounts of their experience with the health service tell us about patients’ experience of accessing general practice?” using secondary analysis of interviews drawn from five studies conducted since 2020. This paper focuses specifically on how patients navigate access systems in UK general practice.

## Methods

The Medical Sociology and Health Experiences Research Group qualitative interview archive held at the University of Oxford contains interviews conducted with patients, family members and carers, collected since the early 2000s for over 100 research projects exploring experiences of a wide range of different health conditions. Projects include studies of arthritis, cancer, neurological diseases, mental health conditions and more recently Covid-19, and explore experience of illness and interactions with health services. While none of the original studies in this archive was directed explicitly to questions about GP access, the narrative and experiential focus of these interviews (which typically cover symptoms, help-seeking, diagnosis and treatment), means that they often include descriptions of patients’ interactions with general practice, including their experiences of seeking and making appointments.

Five studies conducted since 2020 were purposively selected to include people with recent experiences of illness and accessing health services (Table 1). We undertook secondary thematic analysis of interviews conducted with 286 participants (some interviews were follow up interviews with the same participant) from this archive. We extracted data from 124 interviews where access to the GP was discussed (for example where patients described making an appointment with their doctor, phoning the practice or talking to a receptionist to make an appointment etc.).

**Table 1:** the five interview data sets

Project	Short name in this paper	Timespan	Method of recruitment	Reason for inclusion in secondary analysis	Details
Amplifying the voices of people with lived experience to improve understanding, support, treatment and education. Share-to-improve: Long Covid experience (COv-VOICES) Study.	Long Covid	March 2021 - February 2023.	Through various routes, including clinicians, social media, support groups and snowballing to facilitate diversity in experiences and perspectives.	Post-pandemic experiences of long term illness experience, and mixed symptom histories.	27 interviewees (8 Male, 19 Female) UK based adults aged over 18 to understand the lived experiences of people with Long Covid.
Experiences of COVID-19 and recovery: learning from polyphonic voices for communities, policy makers and health and social care providers.	Diverse Covid	December 2020 – June 2022.	Through a variety of routes including clinicians, social media, support groups and snowballing to encourage a wide variety of experiences and perspectives.	Pandemic experiences, reflecting multiple symptoms, presentations including in the period where access was often remote/online; and to include diverse populations.	68 interviewees (24 Male, 43 Female, 1 Non-binary) to learn how adults over 18 from diverse backgrounds experienced COVID-19. The sampling for this project was explicitly designed to capture the experiences of people from Black, Asian and other minority ethnic backgrounds.
Understanding and using family experiences of managing long Covid to support self-care and timely access to services.	Family Long Covid	August 2021-January 2023.	Through social media, clinicians, support groups and snowballing.	Pandemic experiences, reflecting multiple symptoms, presentations to primary care including in the period where access was often remote/online, selected to include people at different stages of the life course.	75 interviewees (19 Male, 56 Female) including children, young people and parents.
Experiences of Urogynaecology Health Services.	UroGyn	January 2021 -June 2022.	Through NHS sites; advertisements, support groups, social media, and advisory panel members; snowball sampling; and working with health advocacy organisations and groups focused on inclusive gynaecological health care.	To include women (who are known to make more use of general practice) and the referral pathway to secondary care	74 interviewees (73 Female, 1 Other) to understand experiences of people over 18 years who have urogynaecological conditions who have used or plan to use urogynaecological health services in the UK.
Older people’s decision-making around knee replacement in the context of multimorbidity.	KneeRep	January 2020 - December 2022.	Through four orthopaedic hospital sites	Interviews were conducted in the period where access was often remote/online - selected to include multimorbidity, people using secondary care, and older people.	42 interviewees (16 Male, 26 Female). Participants (patients or informal carers) were interviewed multiple times to understand the issues and concerns of older people (70–86 years) with severe knee osteoarthritis and multimorbidity making decisions about joint replacement surgery.

This sampling and design follows the ‘formal data sharing ‘mode of secondary analysis described by Heaton [9, 10]. In line with Heaton’s typology, the design can be described as supra analysis in that the aims and focus of the secondary study transcend those of the original research to address new questions.

### Research team

The team was comprised of three experienced qualitative researchers with backgrounds in sociology and anthropology, a junior researcher with a background in sociology, a practising GP and an undergraduate student undertaking a research placement. We ensured reflexivity by having regular meetings to come together and discuss the data, and to compare interpretations. More junior members of the team were asked to set the agenda for the meeting to ensure that power imbalances did not influence the final interpretation.

### Primary datasets

The data sets used are outlined in Table 1.

In the original studies, all interviews were conducted by trained and experienced qualitative researchers. The interviews ranged from 20 to 120 min in duration and were audio recorded and transcribed verbatim. Interviews were conducted in English, Urdu, Hindi and Pashto. Two interviews were conducted, translated, and transcribed by a researcher who spoke Hindi and Urdu. In one interview, the husband of the participant acted as a Pashto interpreter for his wife, and this interview was transcribed by a University approved Interpreting and Translation service.

### Ethical considerations

Informed consent for re-use of de-identified data was available for all studies, and no further ethical permissions were required for the secondary analysis. Nonetheless all team members had appropriate GDPR and information governance training.

### Secondary analysis

The secondary analysis followed a comparative thematic approach, broadly following the steps outlined by Braun and Clarke [11]. The research team read study materials to familiarise themselves with the original study designs, and inclusion and exclusion criteria and scope of each primary study. Two researchers (WA and LF) read and re-read the transcripts, to identify and locate all data items referring to accessing the GP. To augment this manual searching the ‘find’ function in Microsoft Word was used to identify the keywords “GP”, “surgery”, “general practice”, “practice”, “doctor” and “appointment” to ensure that items relating to GP access and use of primary care services had not been missed. Data relating to GP access and achieving access to general practice were coded for retrieval and management in NVivo (version 12). A combination of deductive and inductive coding was used. Deductive codes were generated from the schematic representation (provided in Supplementary file 1) developed from the GP-SUS scoping review [7] which outlined the varied routes to access in general practice (e.g. walk in/face to face access), and inductive codes (e.g. obstacles to access) were developed during the analysis.

The coding team (WA and LF) met weekly to confer and discuss code generation and to challenge each other to enhance the understanding of the emerging codes. The wider research team reviewed and discussed the coding framework and early interpretations to support the development of provisional themes based on grouping and comparing the codes. These themes were then reviewed, through conversations in the team and by searching for and exploring deviant cases. Themes were further refined through the process of interrogation and writing this manuscript to ensure that the analysis accounted for and accurately reflected all the data.

## Results

Our analysis identifies two key dimensions of patient experience, namely ‘loops’ and ‘hurdles’, which together describe how patients encounter repeated cycles and barriers when attempting to access general practice. The analysis presented draws the interviews where GP access was discussed: direct quotes are presented from 18 interviews, chosen to illustrate the findings, but the themes were developed from analysis of 124 interviews (see Table 2). Our secondary analysis uncovered how patients get “lost” or “stuck” when trying to access general practice, and the presence of significant hurdles that prevent patients from gaining timely access to their GP. We have grouped these under two headings (loops and hurdles) and below we explore these themes in more detail.

**Table 2:** Participant demographic table for illustrative quotes* used in this paper

Identifier	Age	Gender	Ethnicity
Family Long Covid 1	38	Female	White British
Family Long Covid 6	59	Female	Black British
Family Long Covid 8	57	Female	White British
Family Long Covid 9	39	Female	Ashkenazi Jewish
Family Long Covid 20	47	Male	White Irish
Family Long Covid 53	22	Female	White British
UroGyn 5	46	Female	Latin Spanish
UroGyn 8	28	Female	White British
UroGyn 20	71	Female	White British
UroGyn 27	41	Female	White British
UroGyn 35	28	Female	White British
Urogyn 45	65	Female	White British
Urogyn 66	48	Female	White British
Diverse Covid 10	37	Male	Black African
Diverse Covid 13	59	Female	Caribbean
Long Covid 20	58	Female	White
Knee Rep 4	78	Female	White British
Knee Rep 8	72	Female	White British

*Quotes were selected from 124 interviews where GP access was discussed, to illustrate themes

### Loops

Patients described situations in which they struggled to access their GP, and where they felt they ‘went round in circles’ or ‘ended up back where they started’. These loops included being asked to “call back tomorrow” or at a later date due to the lack of appointments available, as well as the now ubiquitous online triage systems which patients navigated only to reach a disposition that required them to phone the practice (only to discover that no appointments were available). Patients experiencing Long Covid found the circularity difficult to manage alongside symptoms of fatigue:

When there’s no appointments, they say, “Keep ringing up daily to see if there’s any cancellations.” That’s a lot when I’m having a day where I’ve had a migraine at three o’clock in the morning. I can’t even switch on the light because the light affects my eyes. But now I’ve also got to make a phone call. [Family Long Covid, Interview 53].

and breathlessness:

When you call the GP you’re waiting for ages in a queue and when you get to the queue, when it actually is your turn, you’re struggling to breathe and they can’t hear you, they hang up. So you’ve got to start this all over again, maybe another day because you just can’t do it again. [Family Long Covid, Interview 6]

A ‘triage loop’ was associated with the use of online triage systems. As noted above, this was sometimes entwined with the call back loop, when the result of completing online triage was advice to make an appointment by phoning the GP. This was also an outcome they encountered when using the separate NHS 111 online system. Some patients described delays associated with looping round multiple access systems, in the example below a woman with persistent urinary tract infections (UTI) who experienced a sudden flare-up described using the practice online triage system, phoning the practice, and using NHS111 more than once to get an appointment:

In my GP practice, you can’t call in to book an appointment, you have to fill in this E-consult thing. So, I filled an E-consult one night and […] I didn’t hear anything from them, so I rang, I rang someone the next day and […] they said, “No you will need to [call NHS 111] because we don’t have any availability anyway for today.” So, I rang [NHS 111] and they said, “Yeah you’ll need to be seen within two hours. Ring your GP back and ask them to arrange something for you.” So, I said that to [the receptionist at the practice] and they said, “Okay the doctor will call you.” The doctor didn’t call me all that day. The next day I called 111 and said, “I never heard anything back from them the day before.” and they said, “Okay, we’ll call your GP practice. You don’t need do anything; you’ll hear back from them.” So, the GP practice later that day eventually calls me back, three days after I initially tried to seek treatment. [UroGyn, Interview 35].

When patients missed a promised GP call back, they found themselves back in the loops we have described:

A lot of people get frustrated with how difficult it is to get through on the phone. That is frustrating when you have to keep ringing, and ringing, and ringing, and then you get in a queue, and then you wait for a call back, and then the call back comes when you’re driving and you don’t answer the phone quickly enough, and then, you’ve missed it and that’s really, that’ll do your head in. [Family Long Covid, interview 20]

You call the GP’s surgery, and the GP surgery says your doctor is going to call you, but then it happens that they call you once, and if you miss the call you have to book again […] it is a whole vicious circle. [UroGyn, Interview 5]

Some patients said that continuity of care was important to them and they felt that having a consultation with a GP who they did not know hindered their care experience:

I wanted to stick to the [GP] that sent me into hospital, but when I came out of hospital and had to start again on a sick line [phone queue]. It wasn’t her I got, she was on holiday, it was somebody else I got […] so I just [waited and] stuck with her, and it saved me having to explain everything, because there was one time she was off, and I had to speak to a different doctor, and it was a bit of a waste of phone call, and it was just, it was a tick box. [Long Covid, Interview 20]

However, asking for an appointment with a particular GP might increase delays and require multiple call backs.

### Hurdles

Alongside these ‘loops’, patients described obstacles or ‘hurdles’ that thwarted their access to their GP. A main hurdle was “getting past reception”. This could include navigating telephone triage with the reception staff, or face to face conversations at the reception desk. Patients in the knee replacement surgery study described how waiting in automated phone queues used by many practices was not always possible, due to other commitments including caring, and this was a deterrent to access:

It was so bad, I picked the phone up a couple of times to the surgery to get an appointment to speak to the doctor, let alone see one, and each time I got, I was in a queue. Nine people at one stage. You know, you can’t hang on the telephone because it’s costly. And so, I gave up in the end and I just put up with it [Knee rep, Interview 4].

Others found that phoning as soon as phone lines opened was difficult:

You’ve got to ring up at eight o’clock of a morning to get an appointment. Well, I’m sorry but I’ve been awake half the night in pain sweating [with a UTI flare-up], you know, I’m not, I’m not up at eight o’clock in the morning, the mornings are my worst, absolute worst, so invariably I don’t get an appointment. [UroGyn, Interview 45]

Patients expressed discomfort about having to share confidential and sensitive details about their condition with reception and administrative staff when attempting to secure an appointment (e.g. UroGyn, Interview 8). Patients felt that this triage was intrusive, a form of gatekeeping and ultimately duplicated information that the doctor needed to know:

you have to get past the Rottweiler – [the] receptionist to make an appointment with the doctor now, … who wants to know everything. So I get quite graphic some days. [UroGyn, Interview 66]

Even if it’s not Covid, you tell the receptionist about your illness and everything and your symptoms, and … this is personal, do they need to know about this? Or should the GP know about this first? Instead of passing to the receptionist, and then the receptionist is going to decide to talk to the GP. To what extent does [the receptionist] have knowledge, the capacity, to be able to speak, to say okay, you’re going to see the GP or not. Basically, what I feel like is we should speak to the GP, and the GP would hear us, and they would say whether we needed to see him or not. [Diverse Covid, Interview 10]

### Patient responses to loops and hurdles

Patients described strategies they used to deal with the loops and hurdles we have described. This included trying to persuade the reception staff, and emphasising symptoms or need in an attempt to get an appointment designated for urgent or emergency consultations. Some patients admitted that they had heard others “tell fibs and say it’s worse than it really is” [Family long Covid, interview 8] to get this kind of appointment. Another patient asserted their professional title when negotiating access to try to get an appointment:

[I said] It’s not “Mrs”, it’s “Doctor”.” […] Suddenly her whole tone changed, I am sure again it was not intentional, but suddenly it was much more acknowledging what I’m saying and not just trying to, you know, follow the policies, and so, I get “there is an emergency doctor, he can call you. [Family Long Covid, Interview 9]

A patient in the urogynacology study described how, having initially not being offered an appointment, she persisted, being “a bit of a pain” to get one:

It’s not in my nature to just accept it, so I didn’t. […] I would’ve made an appointment again two days later and been a bit of a pain until I got a reaction. But a lot of people would, wouldn’t think that, they would just, oh okay, well, they they’ve told me this, so obviously I just have to live with this. [UroGyn, Interview 27]

Familiarity with the practice access system, for example knowing when a preferred GP was available could help ensure continuity of care and support attempts to get an appointment:

I knew to ring and ask for the same GP and I knew which days of the weeks she works, and also with a long-term condition you can leave it a few days if you need to wait to speak to them because you’re still going to have [the problem] in a few days’ time. [Family Long Covid, Interview 1]

But these strategies were not available to all patients. For example, one patient felt that their migrant status and “lack of knowledge” of the general practice system made it especially difficult to navigate NHS services [Family Long Covid, Interview 9]. Another patient mentioned that people with reduced capacity due to illness or disability might find access more difficult:

I think like for me, I can work my way around the system. What about if I was somebody who was, you know, probably more housebound, had other complications, and was worried about the same thing I was? [Diverse Covid, interview 13]

Covid lockdown limits on face to face consultations and greater use of telephone access was also referred to in the Covid studies as making access to GP appointments difficult, but was also mentioned in the urogynaecology study:

I think Covid is what’s made everything so difficult because you can’t see a doctor anymore. Everything you do is on the telephone and that’s even if you can get a telephone appointment. The last time I needed to speak to a doctor, I had to wait three weeks for a telephone appointment. I think healthcare has gone down the drain to be perfectly honest. [UroGyn, Interview 20]

Post-pandemic, patients contended that demand for GP services was still limiting access, especially for routine follow up care:

They’re not offering any [appointments] I wrote… and said I was due for a diabetic review in September and I wrote three or four times and all I got back was, well they’re “looking at how we are going to achieve all those”. [Knee rep, Interview 8]

Our secondary analysis of these five interview datasets provides additional detail about some of the loops and hurdles patients experience when attempting to access GP appointments, for Covid and for non-Covid related conditions, and their responses to these access challenges. Some variation in experiences was evident, for example, patients with fewer resources and those who were less familiar with the access systems appeared to report greater difficulty overcoming hurdles, but the nature of the dataset does not allow systematic comparison across all demographic groups (we consider this further in the limitations section below). That said, it is clear that loops and hurdles are a common feature of patients’ access experiences. The final section of our paper discusses these findings in the light of previous work and other research and policy.

## Discussion

### Summary of our findings

Building on our published scoping review [7], the secondary analysis presented here has allowed us to ‘zoom in’ on the complexity of patients’ experiences, to examine their difficulties accessing their GP. From the practices’ perspectives, systems such as online triage, or redirection of patients to other care providers who are not GPs, are implemented to manage demand, often, as shown in our scoping review, with the intention of improving efficiency [7]. But some patients experience these systems as inhibiting access. The loops and hurdles they described highlight where access is most difficult and the frustrations linked to the circularity of phone and online triage systems. Some patients have strategies and resources that they can deploy to overcome these barriers to access. These include discursive strategies such as the use of the professional title, or knowing how to persist when asking for an appointment.

### Strengths and limitations

We analysed patient experiences to augment our understanding of access to general practice since 2020. This adds to evidence about the challenges patients encounter in getting to see their GP. The archived interview data used for this research comprised recent interviews with people from different age groups and a variety of medical conditions. These data allow us to understand different patient access experiences, including during the Covid-19 pandemic. However, they do not reflect the most recent structural and organisational changes in the way that access is organised in general practice [12]. Our secondary analysis was limited by the scope and content of the original studies and research questions: the included studies were not designed specifically to examine GP access, and demographic information was not consistently available or recorded in standardised ways across each study and this limits our ability to explore differences by patient characteristics such as disability, socioeconomic status or ethnicity in a systematic way. We acknowledge that patients with Long Covid are likely to have experienced difficulty with access, given they were accessing care for a poorly understood and initially unrecognised condition at a time when access was already more difficult to obtain [13, 14]. Some of the studies intentionally oversampled marginalised or vulnerable populations, while others were dominated by particular groups. While the findings reflect diversity, women’s experiences are over-sampled in the composite dataset because we included the UroGyn study [15] and because women outnumbered male respondents in all the data sets. Similarly, the quotes used are predominantly from White participants who dominate the samples, notwithstanding the attempts of the included studies to diversify recruitment. We note too that the use of an informal family member to interpret in one (Pashto) interview while adding to the sample diversity is recognised as a methodological limitation in both medical consultations [16] and qualitative health research [17]. However, as with all qualitative research, the aim here is not for statistical generalisability but analytical richness that offers transferable ideas and insights.

There are undoubtedly gaps in understanding that will be better filled by further primary research, and this study should sit alongside further research exploring and attempting to understand patient experience in depth.

### Comparison with other literature

Patients who have language challenges or poorer ability to describe symptoms are known to be less likely to get appointments [18, 19] and in our secondary analysis patients with Covid who struggled with breathlessness, and those who lacked language skills and detailed knowledge of the health system were at a disadvantage. The ability to call back repeatedly or spend a long time in a telephone queue could aid getting an appointment, but not all patients could do this. These kinds of social, cultural and economic capital [20] can exacerbate inequalities related to access. The use of online and digital systems is also marked by inequalities: women, those with higher education levels, and those with long-term health conditions make greater use of online health services [21, 22]. Increased reliance on NHS 111 online and non-clinical telephone triage was also highlighted as a cause of systematic health inequalities in the pandemic [23]. Our secondary analysis demonstrates how such digital systems can act as barriers to access.

Previous research examining access to primary mental health care for ‘hard-to-reach’ groups has shown that patients often need to navigate complex networks of services [24]. Our findings mirror this, highlighting how patients with different symptoms and conditions repeatedly engage with multiple systems and processes to secure access to a GP.

Existing theoretical frameworks such as ‘Access as Human Fit’ [25] and Levesque’s model [26] emphasise the interaction between system accessibility and patient capability. Our findings augment these frameworks by showing that contemporary access systems generate recursive ‘loops’ and ‘hurdles’ that require patients to deploy resources, such as time, knowledge, persistence, to successfully access care. Our findings highlight the dynamic and iterative nature of access, rather than the linear pathway often imagined in health policy and access system design.

### Implications for practice and research

It is not clear that the solutions proposed by policymakers will address the barriers to access described in the UK Government’s 10-year plan for the NHS. Our findings confirm the conclusions of a recent qualitative interview study which also argued that there is a mismatch between what patients want and the solutions proposed in the NHS 10 Year Plan [27]. A heavy focus on self-care, digital tools and diversion to other health professionals such as pharmacists [3] may, for many patients simply add to their experiences of “going round in circles”. Research focused on patients is needed, especially to investigate the links between the access systems used in general practices and patient health outcomes. UK policy is increasingly directed to access solutions involving telephone triage and digital-first access systems, especially the digital systems that made ubiquitous by the Covid-19 pandemic and as we have shown these may increase barriers to access, and fuel patient and public dissatisfaction. Our work points to impacts on patients’ experience and we will need a better understanding of this when moving forward with new access initiatives.

## Conclusions

Secondary analysis of existing datasets about healthcare experience can provide useful insight about how patients experience access to general practice. The study contributes to our understanding, and the findings are an important focus for consideration if services are to be delivered in a way that works for patients.

## Supplementary Information

Supplementary Material 1.

## Data Availability

The datasets analysed for this paper are available from the corresponding author on reasonable request.

## Abbreviations

GP: General practitioner
UK: United Kingdom
GDPR: General Data Protection Regulation
NHS: National Health Service
