# Supplementary figures and images for: “Loops and hurdles”: secondary analysis of patient interview data to explore the experience of patient access to UK general practice
Source: BMC Prim Care. 2026 May 16;27:257. doi: 10.1186/s12875-026-03376-5 (PMC13348686; doi:10.1186/s12875-026-03376-5)

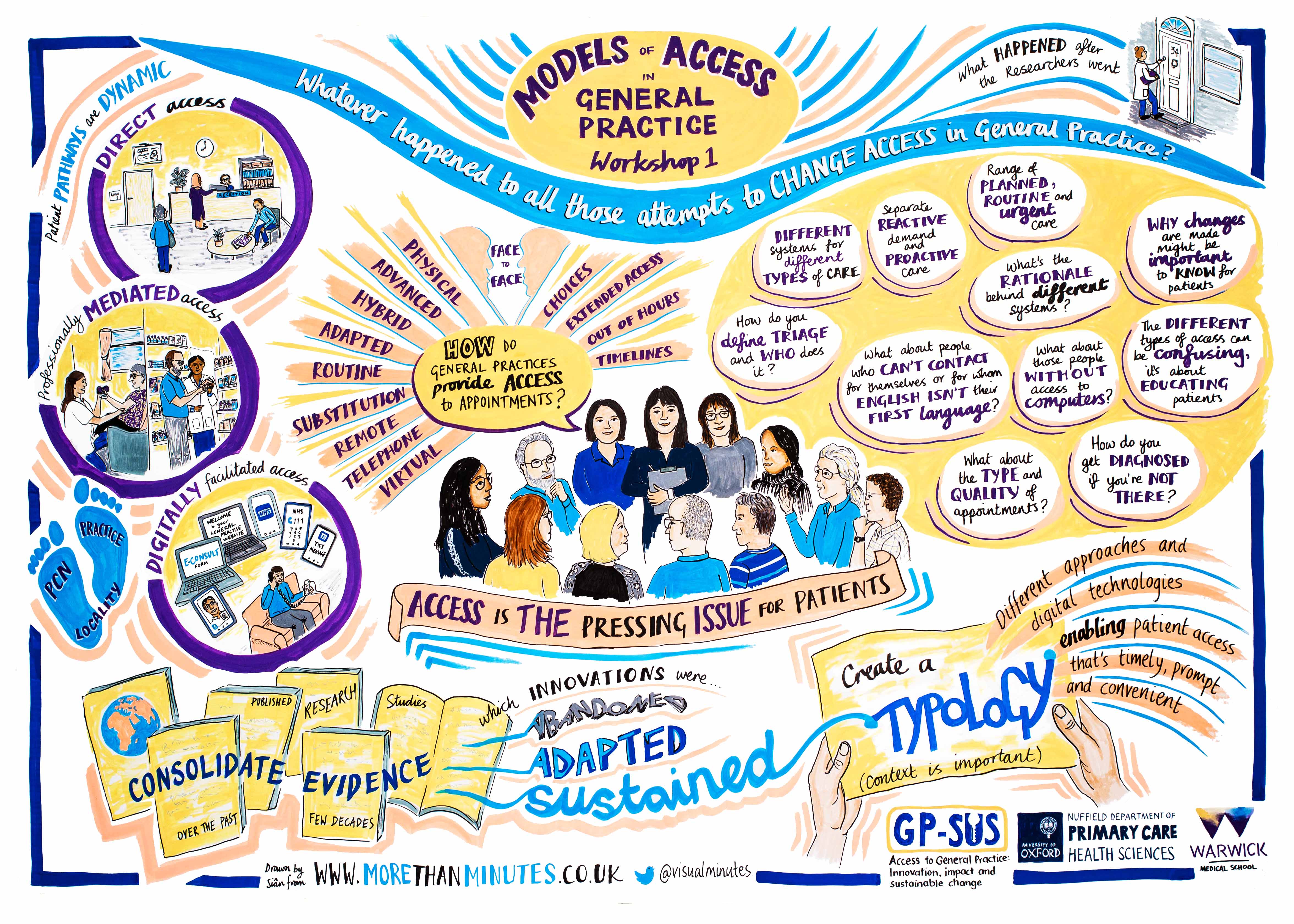

Supplement: Supplementary file 1 — Supplementary Material 1. [file 12875_2026_3376_MOESM1_ESM.jpg]
